# Seven Conformations of the Macrocycle Cyclododecanone Unveiled by Microwave Spectroscopy

**DOI:** 10.3390/molecules26175162

**Published:** 2021-08-26

**Authors:** Ecaterina Burevschi, M. Eugenia Sanz

**Affiliations:** Department of Chemistry, King’s College London, London SE1 1DB, UK; ecaterina.burevschi@kcl.ac.uk

**Keywords:** conformational flexibility, rotational spectroscopy, large ring, cycloketone, intramolecular interactions

## Abstract

The physicochemical properties and reactivity of macrocycles are critically shaped by their conformations. In this work, we have identified seven conformations of the macrocyclic ketone cyclododecanone using chirped-pulse Fourier transform microwave spectroscopy in combination with ab initio and density functional theory calculations. Cyclododecanone is strongly biased towards adopting a square configuration of the heavy atom framework featuring three C–C bonds per side. The substitution and effective structures of this conformation have been determined through the observation of its ^13^C isotopologues. The minimisation of transannular interactions and, to a lesser extent, HCCH eclipsed configurations drive conformational preferences. Our results contribute to a better understanding of the intrinsic forces mediating structural choices in macrocycles.

## 1. Introduction

Macrocycles are molecules containing twelve-membered or larger rings, used in drug discovery [1], catalysis and materials [2]. Their stability, reactivity and physicochemical characteristics are intimately related to their structural properties, specifically to the conformations they adopt and their intramolecular forces. Conformational analysis of macrocycles is crucial to characterise their ability to interact with other molecules, predict possible changes in their configurations and design modified macrocycles with pre-selected conformations. However, investigation of the intrinsic conformational behaviour of macrocycles is particularly challenging because they are highly flexible and present serious difficulties for their experimental analysis.

Studies of cycloalkanes C_n_H_2n_ have mainly focused on small rings (up to 7 atoms) and, to a smaller degree, on medium rings (8 to 11 atoms) [3,4,5,6,7,8]. Torsional and angle strain determine the conformations of small cycloalkanes with n = 3–5. Due to ring constraints, ∠CCC angles are reduced to values below 109.5°, and, in four- and five-membered rings, large amplitude motions, such as ring-puckering and pseudorotation, are common to minimise strain. Torsional and angle strain are basically removed in six-membered cyclohexane (n = 6), which adopts a chair conformation wherein bond and torsional angles are close to their optimal values for tetrahedral carbons, and all C–H bonds are staggered, avoiding eclipsed interactions [9]. However, in cycloheptane (n = 7), there is torsional strain due to eclipsing interactions between H atoms, like in smaller rings, and also steric strain caused by transannular repulsive interactions, which are characteristic of medium-sized rings [10]. Cycloheptane is therefore not strain-free and experiences pseudorotation [10,11,12].

Unlike small rings, medium-sized rings have less angle and torsional strain, but they exhibit a high level of steric strain. This is often due to the unfavourable transannular interactions involving the hydrogen atoms pointing inside the ring. The decrease of angle and torsional strain and the increase in ring flexibility result in medium-sized cycloalkanes generally adopting more than one conformation. Structural studies on cyclooctane in the gas phase revealed two conformations being present, a predominant boat-chair conformation and a crown conformation [13]. Similarly, cyclononane [14] and cyclodecane [15] show a more favoured conformation and a few less abundant ones. The last member of the series, cycloundecane, has been found to present two conformations in solution at low temperature [16].

In macrocyclic cycloalkanes, with 12 atoms and above, there is a considerable reduction in strain [12]. For cyclododecane, studies by electron diffraction [17], X-ray crystallography [18] and NMR [19] identified a square configuration, with three C–C bonds per side, as the predominant one. In this conformation, the carbon chain adopts a structure similar to an aliphatic chain, with an anti-zigzag configuration and staggered hydrogens [18]. Whilst there was evidence of other more distorted structures being present, they could not be identified. Experimental investigations on larger cycloalkanes are considerably sparser. To our knowledge, there is only an X-ray structural determination of cyclotetradecane [20].

Elucidating the forces determining the preferred conformations of 12-membered rings is the basis to understanding the conformational features of larger macrocycles. Structural experimental techniques such as NMR, X-ray and electron diffraction are still limited in their ability to distinguish between multiple conformations, despite advances in their capabilities [21,22,23]. Because of this, macrocycle derivatives have been investigated in some cases, as substituents helped crystallisation or removed some of the conformational flexibility. The studies of derivatives are also important, as modifications of the forces involved in modelling the conformational landscape shed light on their relative significance [24,25,26,27,28,29,30].

Rotational spectroscopy is arguably the most powerful structural technique for conformational identification because each conformation produces a distinct rotational spectral pattern indicative of its mass distribution and therefore, can be unequivocally identified. The only requirement is that the target molecule has a permanent dipole moment. Broadband spectral collection, typically of several GHz, is essential for decoding the complex spectrum of multiconformational molecules [31,32,33,34,35]. Using broadband rotational spectroscopy, we have recently analysed the conformational behaviour of cyclooctanone, a medium-size cyclic ketone [36]. We identified three conformations: a strongly prevalent boat-chair (analogous to that of the unsubstituted cycloalkane), a twisted boat-chair and another boat-chair conformation. The presence of transannular interactions was the driving force behind conformational behaviour. 

In this paper, we apply broadband rotational spectroscopy to the conformational study of cyclododecanone, the smallest of the large-ring ketones, as a first step to advance our understanding of the behaviour of large rings. Cyclododecanone is used in the synthesis of complex natural compounds containing macrocycles in their structures, such as the anticancer reagent rosphellin [37,38], and of macrocycles used in musk fragrances, like muscone, exaltolide and others [39,40,41,42,43]. It has been studied in condensed phases by X-ray diffraction [44] and by low-temperature ^13^C NMR and ^1^H NMR [27], where it was found that it exists as one conformer that maintains the square configuration reported for cyclododecane. However, there are no previous studies of cyclododecanone in the gas phase, wherein its intrinsic conformational preferences, unaffected by intermolecular forces, can be revealed.

Cyclododecanone is expected to have a richer conformational landscape than cyclooctanone due to its larger size and different interplay of intermolecular forces directing conformational behaviour. Our results confirm these expectations. We have identified seven conformations of cyclododecanone, of which a square configuration is significantly more abundant, and characterised their non-covalent interactions. A combination of transannular H∙∙∙H interactions and eclipsed HCCH configurations determine the conformational stability of cyclododecanone.

## 2. Results and Discussion

### 2.1. Rotational Spectrum

To aid spectral searches, the lower-energy conformations of cyclododecanone were predicted by mapping its potential energy surface using the semiempirical method AM1 within the program HyperChem [45] and the conformer sampling program CREST [46]. Overall, 18 distinct conformers were calculated within 1000 cm^−1^ (see Table 1 and Table S1). All of them have a sizable *μ_c_* dipole moment component and are expected to show a predominantly *c*-type spectrum.

The 2–8 GHz broadband rotational spectrum of cyclododecanone showed a set of lines significantly more intense than all others. Considering the theoretically predicted spectral patterns, they were easily identified as *c-*type *J* + 1 ← *J* transitions. From a preliminary fit of these transitions and subsequent prediction of the spectrum, *a-* and *b-*type lines belonging to the same species were observed with much lower intensity and measured. A total of 81 transitions (see Table S2 in Supplementary Materials) were fit to the semi-rigid rotor Watson Hamiltonian in the A reduction and III^l^ representation [47] using Pickett’s program [48], yielding the rotational and centrifugal distortion constants of column 1 in Table 2. Comparison with the theoretical rotational constants identified the observed species as conformer **I** of cyclododecanone. Due to the high intensity of the lines, the ^13^C isotopic species were also observed at the predicted frequency shifts with respect to the parent species and assigned. Their observed transitions were fit to the same Hamiltonian [46] as the lines from the parent species (Tables S3–S14), and the determined rotational constants are collected in Table S22. The ^18^O isotopologue was searched for, but it could not be observed, probably due to its lower natural abundance (0.2%) compared to the ^13^C (1.1%).

Removing the lines belonging to conformer **I** allowed for the observation of less intense lines, revealing the true density of the spectrum and suggesting that more conformations were present. Using the automated spectral assignment of PGOPHER [49,50] in spectral searches, six additional conformations of cyclododecanone were assigned. The automatic assignment procedure in PGOPHER starts by choosing some parameters to refine, and a range for all or some of them; a few predicted transitions to attempt a preliminary fit, and a search window around them; and some additional transitions within a specified acceptance window to be added to the initial fit and confirm it. We selected the three rotational constants as parameters to refine with an initial floating range of 20 MHz, a set of three *c*-type transitions with different *K*_-1_ values to try an initial fit, and further *c*-type transitions to be searched for and included in the fit if a first satisfactory match was achieved.

All the conformations showed primarily *c-*type spectra, and their transitions were fit to the same Hamiltonian used for conformer **I** for oblate tops and A reduction and I^r^ representation [47] for prolate tops. The resulting rotational and centrifugal distortion constants are shown in Table 2. The measured transitions are included in Tables S15–S21. The conformations are identified as conformers **II**, **III**, **IV**, **V**, **VI** and **VII** from the close values of the theoretical and experimental rotational constants. Higher energy conformations were searched for, but they were not observed in the spectrum, probably due to their low population in the molecular jet.

**Figure 1:**
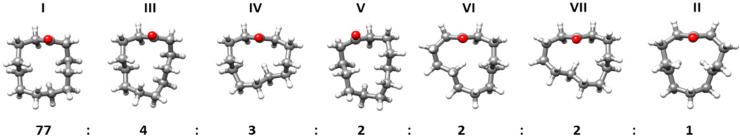
Relative conformational abundances of the seven observed conformers of cyclododecanone.

We have estimated the relative abundances of the observed conformers by comparing common *c-*type transitions and considering that the relative abundance of a conformer is directly proportional to the intensity of its transitions and inversely proportional to the square of the corresponding dipole moment component. We used the expression $\frac{N_{1}}{N_{2}}=\frac{I_{1}\mu_{2}^{2}}{I_{2}\mu_{1}^{2}}$, where *N* is the number density of the corresponding conformer; *I* is the observed line intensity, and *μ* is the theoretical *c* dipole moment component. The values obtained are **I**:**III**:**IV**:**V**:**VI**:**VII:II** = 77:4:3:2:2:2:1, respectively (see Figure 1). Conformer **I** is significantly more abundant than the others, as expected from its spectral intensity. It is 19 times more abundant than the second-most abundant conformer. The other six conformers are much more similar in abundance, with a range of 1–4 between them.

The theoretical rotational constants predicted by B3LYP-D3BJ and MP2 agree well one with another and are very close to the experimental values, with average deviations of 0.6% for B3LYP-D3BJ and 1.2% for MP2 (see Table S22). Both methods predict conformer **I** as the global minimum and an energy gap with the next conformer of approximately 450 cm^−1^, which is consistent with experimental observations. The observed conformers are calculated to be those lower in energy by both theoretical methods, and although the energy ordering is not exactly the same, the differences in values are small. The estimated relative populations based on the theoretical values of the B3LYP-D3BJ values of the Gibbs free energies predict conformer **I** to be more abundant than the other conformers, and a gap in abundance between the observed conformers and those predicted at higher relative energies. However, they do not fully explain the observed conformational abundances probably due to the existence of collisionally induced relaxation in our supersonic jet from higher-energy conformers to lower-energy ones.

### 2.2. Experimental Structure of Conformer I

**Figure 2:**
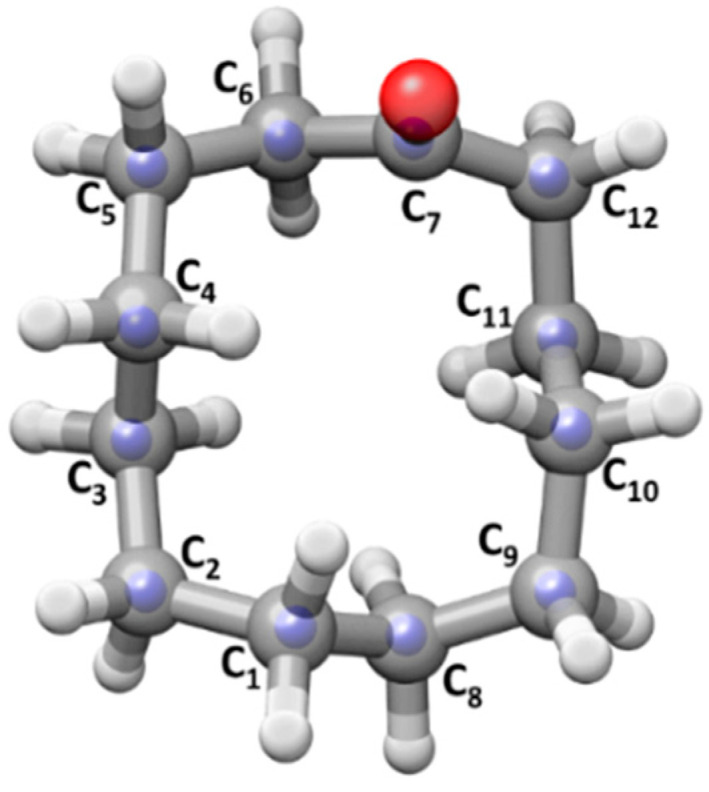
*r*_s_ structure of conformer **I** (blue spheres indicating the position of the C atoms) overlapped with B3LYP-D3BJ/6-311++G(d,p) (grey framework), including atom labelling.

The observation of the ^13^C monosubstituted isotopic species has allowed the determination of the coordinates of the carbon atoms in the principal inertial axis system using Kraitchman’s equations (see Table S23) and the subsequent calculation of the substitution structure *r*_s_ [51] of the carbon framework. The experimentally determined bond lengths (*r*), bond angles (∠) and dihedral angles (τ) of cyclododecanone are shown in Table 3 and Figure 2.

There is quite a disparity in the values of the bond lengths determined by the *r*_s_ structure, which could be attributed to the small coordinates of some of the carbon atoms. The most obvious example is C_7_, whose *c* coordinate was fixed to zero as the Kraitchman calculation returns an imaginary value. The bond lengths involving C_7_ are abnormally small considering that the average sp^3^ C–C distance is 1.54 Å. The atoms C_2_, C_4_ and C_5_ also have small coordinates affected by large uncertainties, and this is reflected in the *r*_s_ values of the bond lengths in which they participate. The uncertainties in the coordinates also result in an unreasonably large value of 125.2(37)° for the bond angle ∠C_12_C_7_C_6_. The other bond angles are mostly similar to those of the B3LYP-D3BJ equilibrium structure (Table 3). However, some of the *r*_s_ dihedrals involving C_2_ and C_7_ display differences of up to 5° with *r*_e_ and *r*_0_ values.

The *r*_0_ structure was obtained from the fit of the 39 experimental moments of inertia to determine 11 bond lengths, 9 bond angles and 6 dihedral angles involving the carbon atoms. The structural parameters that were not floated were fixed to their B3LYP-D3BJ values. The *r*_0_ bond lengths are close to the expected values for single C–C bonds, ranging between 1.52–1.54 Å. The bond angles roughly span 111–115°, and they are similar to those determined for cyclooctanone [36]. The majority of the dihedral angles are close to those of 60°, 180° and −60°, reducing torsional strain. For comparison, cyclooctanone had two dihedral angles with values of 105.1(2)° and 139.7(4)°, indicative of higher torsional strain. There is a good agreement between the *r*_0_ and the *r*_e_ structure.

The previously reported X-ray structural data on cyclododecanone shows atypical values for bond lengths and bond angles varying between 1.45–1.62 Å, and 108–119° [44], respectively, which the authors attribute to problems with X-ray crystal structure refinement. Therefore, we are not comparing our data to their reported parameters. Cyclododecane has been shown by electron diffraction [17] and X-ray crystallography [18] to predominantly adopt a square conformation like that reported here for cyclododecanone. However, the above studies on cyclododecane only provide average structural parameters, which does not allow for a proper comparison with our data.

### 2.3. Conformations and Intramolecular Interactions 

The conformational landscape of cyclododecanone can be understood in terms of the various types of strain at play. Because cyclododecanone is a large ring, it is anticipated to have low torsional and angle strain. Normally angle strain does not contribute strongly to conformational energy differences [12]. Considering B3LYP-D3BJ cyclododecanone structures, which are expected to be very close to the experimental ones, ∠CCC bond angles are very similar among all the observed conformations, in the range 112–117°. Torsional strain is expected to introduce larger differences between conformations. However, dihedral CCCC angles are mostly close to the idealised angles of 60, 180 and −60°. Marginally bigger differences from ideal angles are observed in the least abundant conformers, such as conformer **II** with torsional angles of −138.1 and 146.2°, and conformer **VII** with an angle of 134.3°.

**Figure 3:**
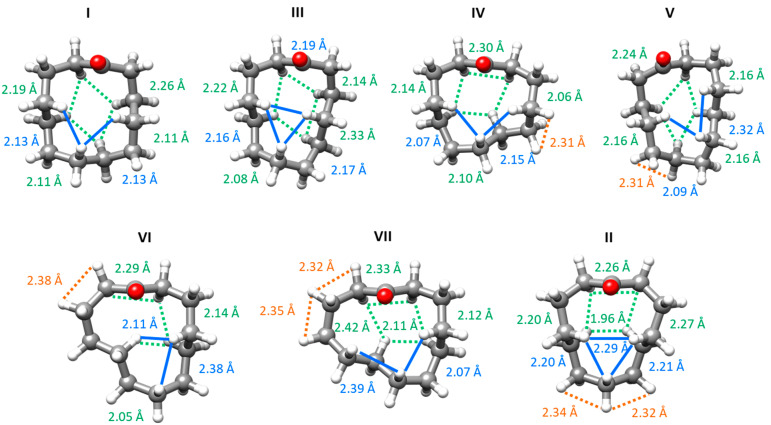
Observed conformers of cyclododecanone showing the transannular interactions, indicated in blue (above the ring, on the side of the carbonyl group) and green (below the ring, on the opposite side to the carbonyl group). The HCCH-eclipsed interactions are indicated in orange. The B3LYP-D3BJ values of the interaction distances are shown in close proximity to the corresponding interaction and in the same colour.

Transannular interactions, or steric strain, occur in all conformations of cyclododecanone. These are due to the interactions of the H atoms directed to the interior of the ring. The majority of conformers have seven intra-annular H atoms, three on the same side of the ring as the carbonyl group and four on the other side. The exception is conformer **VI**, with six intra-annular H atoms. These hydrogens are generally at distances shorter than the sum of the van der Waals radii of the hydrogen atoms (2.40 Å) [52,53], giving rise to a varying number of transannular interactions (see Figure 3). In addition, some of the conformers also show HCCH-eclipsed configurations, at distances of 2.31–2.38 Å and dihedral angles of 32–49°. For comparison, eclipsed interactions occurred in all observed conformers of cyclooctanone at slightly shorter distances of 2.27–2.28 Å and dihedral angles of 13–22° [36]. The effect of eclipsed configurations is therefore expected to be less relevant for cyclododecanone, but their combination with transannular interactions drives conformational stabilities. The strong preference for conformer **I** can be explained by its lack of eclipsed configurations and the fact that it shows six transannular interactions at distances ranging between 2.11 Å and 2.26 Å. Conformer **III**, the second in abundance, presents seven transannular interactions with distances between 2.08 Å and 2.33 Å. All of the other conformers present both transannular and eclipsed interactions (see Figure 3). The least abundant conformer **II** shows the shortest transannular interaction by far (1.96 Å), out of seven overall, and two eclipsed configurations.

In all the conformations of cyclododecanone, the carbonyl bond is roughly perpendicular to the plane of the ring. This angle is reduced from that in cyclooctanone, wherein it was roughly 120 degrees. This behaviour is consistent with expectations of changes in this angle in moving from smaller to larger rings. In small rings with 3–5 atoms, the angle between the carbonyl group and the plane of the ring is around 180 degrees. As the size of the ring increases, the angle becomes more and more acute, until for very large rings, the carbonyl bond could even point inside the ring, forming an angle of zero degrees. These different configurations respond to the smaller strain expected in large rings and the possibility of interactions of the carbonyl oxygen with the H atoms pointing towards the inside of the ring.

The conformational preferences in cyclododecanone are strongly biased to conformer **I**, which is 19 times more abundant than the second-most abundant conformer. This strong preference for one conformation was also observed in cyclooctanone, but with a more marked predominance of the most abundant conformer, which was 44 times more abundant than the second one. In comparison with cyclooctanone, the number of lower-energy conformations is larger in cyclododecanone, and the gap between the global minimum and the next conformer is energy is smaller. Therefore, in cyclododecanone the population is spread over a larger number of structures, decreasing the span of relative abundances. Further studies of even larger cycloketones will be able to ascertain whether there is a general trend wherein the dominance of one conformation decreases as the size of the ring increases.

Although there is less strain in cyclododecanone in comparison with cyclooctanone, the same intramolecular transannular H∙∙∙H and eclipsed HCCH interactions control conformational preferences. Both cycloketones are even-membered cycles, which generally have received more attention than odd-membered ones, and have a higher number of theoretical and experimental investigations devoted to them. Odd-membered cycles are more difficult to synthesise and more reactive. Assessing their conformational behaviour in light of that of even-membered cycles will improve our understanding of their reactivity and physicochemical characteristics.

A comparison of the behaviour of cycloketones with other large rings bearing different functional groups will be interesting to determine the functional group influence on the conformational landscape and intramolecular interactions. For example, rotational studies of the 12C4 and 15C5 crown ethers also seem to point to a lower predominance of one conformation with larger ring size. Although the lower-energy conformations of bare 12C4 could not be observed because they have no dipole moment, theoretical calculations predict a similar behaviour to cyclododecanone with a highly favoured lowest-energy conformation [54]. For bare 15C5, nine conformations were observed with closely predicted relative energies [35,55,56]. For these systems, the relevant intramolecular forces were found to be attractive C–H∙∙∙O and repulsive O∙∙∙O interactions, very different from cycloketones due to the high number of ether groups. The use of rotational spectroscopy has been invaluable to reveal conformational behaviour and the intrinsic forces mediating structural preferences. 

## 3. Materials and Methods

The broadband spectrum of cyclododecanone (Sigma-Aldrich, >99%) was recorded using our chirped-pulse Fourier transform microwave spectrometer at King’s College London; the spectrometer operates in the 2–8 GHz frequency range and has been previously described [57,58]. Cyclododecanone, heated at 417 K in our bespoke heating nozzle, was seeded in neon at 5 bar and conducted to the vacuum chamber, where the molecules expanded adiabatically forming a supersonic jet. The molecular pulse was polarized by four chirped microwave pulses of 4 µs duration each. After each microwave pulse, the emission signal was collected as free induction decay (FID) for 20 µs, amplified and stored in a fast oscilloscope in the time domain. A fast Fourier transform algorithm was used to convert the time domain spectrum to the frequency domain. The final spectrum has 1.5 M FIDs.

The potential energy surface of cyclododecanone was explored using the semiempirical method AM1 within the conformational search function implemented in the program HyperChem [45] and the conformer sampling program CREST [46]. The first 200 conformations generated by HyperChem were optimised at the B3LYP-GD3BJ/6-311++G(d,p) level of theory, returning 16 distinct conformers within 1000 cm^−1^. CREST generated 176 conformers overall, which were optimised using the same method. Together both methods generated 18 distinct conformations within 1000 cm^−1^. The structures predicted by both methods agree well, except for conformers **IX**, **XI**, and **XV**, which were predicted only by HyperChem, and conformers **VIII** and **X** which were predicted only by CREST. The resulting geometries have been further optimised at the MP2 level of theory with the 6-311++G(d,p) basis set, and the their zero-point corrections were calculated using both methods to confirm that the predicted conformers are real minima and not saddle points. The predicted conformers within 1000 cm^−1^ are presented in Table 1 and Table S1. All of them have a sizable *µ_c_* dipole moment component and are expected to show predominantly *c*-type spectrum.

## 4. Conclusions

Seven conformations of cyclododecanone have been unambiguously identified by broadband rotational spectroscopy. There is a strong preference for the macrocycle to adopt a conformation with a square configuration of the heavy atom framework featuring three C–C bonds per side. This conformer is 19 times more abundant than the second-most abundant conformer and 77 times more abundant than the least-abundant one. The minimisation of transannular interactions and, to a lesser extent, HCCH eclipsed configurations, drive conformational preferences. Rotational spectroscopy, specially including broadband operation, is an ideal method to disclose the conformations of macrocycles and other flexible molecules and get insight on the intramolecular forces at play.

## Figures and Tables

**Table 1:** Ab initio ^[a]^ spectroscopic parameters for the conformers cyclododecanone within 700 cm^−1^.

	I		II		III		IV		V		VI		VII	
	B3LYP	MP2	B3LYP	MP2	B3LYP	MP2	B3LYP	MP2	B3LYP	MP2	B3LYP	MP2	B3LYP	MP2
*A*^[b]^ (MHz)	872.3	888.5	896.1	918.6	967.3	979.6	858.0	880.7	988.5	1002.0	914.3	925.0	943.7	951.8
*B* (MHz)	691.1	693.2	693.2	697.0	632.2	641.2	740.9	741.3	630.1	635.2	673.7	680.3	682.2	687.6
*C* (MHz)	447.0	453.0	450.7	459.1	450.4	459.4	461.2	468.1	445.6	451.7	442.3	447.9	453.3	458.3
*μ_a_* (D)	0.9	0.8	0.8	0.6	0.9	0.7	−0.2	−0.2	1.3	1.1	0.3	0.2	−0.1	−0.1
*μ_b_* (D)	0.2	0.2	0.2	0.4	−0.6	−0.5	0.5	0.5	0.4	0.4	−0.3	−0.3	−0.3	−0.2
*μ_c_* (D)	−2.6	−2.4	−2.3	−2.2	2.6	2.4	2.4	2.2	−2.5	−2.3	−2.4	−2.2	2.4	2.2
Δ*E* ^[c]^ (cm^−1^)	0.0	0.0	469.1	516.4	461.6	401.9	583.8	574.0	495.3	452.6	645.6	604.9	698.0	726.3
Δ*E*_0_^[d]^ (cm^−1^)	0.0	0.0	446.4	527.8	455.8	443.3	575.0	585.1	590.6	508.3	592.1	572.0	611.2	673.8
Δ*G* ^[e]^ (cm^−1^)	0.0	0.0	269.7	566.5	537.7	579.2	521.7	649.4	780.9	663.3	583.4	538.2	594.6	696.4

^[a]^ B3LYP-D3BJ and MP2 with 6-311++G(d,p) basis set. ^[b]^ *A*, *B*, *C* are the rotational constants; *μ_a_*, *μ_b_* and *μ_c_* are the electric dipole moment components. ^[c]^ Relative electronic energies. ^[d]^ Relative electronic energies including the zero-point correction. ^[e]^ Gibbs free energies at 417 K.

**Table 2:** Experimental spectroscopic parameters of the seven observed conformers of cyclododecanone.

Parameter	I	II	III	IV	V	VI	VII
*A*^[a]^ (MHz)	865.65313(23) ^[f]^	894.30343(70)	966.10552(37)	868.61009(42)	987.56269(41)	915.21738(48)	942.67936(52)
*B* (MHz)	700.47778(27)	677.77615(98)	638.54650(36)	739.83405(53)	634.23052(50)	677.88681(41)	684.66240(45)
*C* (MHz)	449.17010(22)	451.3106(34)	453.92123(43)	463.7153(46)	447.67053(47)	444.2827(29)	454.18205(29)
∆*_J_* (kHz)	0.1378(71)	0.080(17)	0.0295(52)	0.144(10)	0.0305(58)	-	-
∆*_JK_* (kHz)	−0.336(25)	-	-	−0.275(30)	-	-	-
∆*_K_* (kHz)	0.205(19)	-	-	-	-	-	-
*δ_J_* (kHz)	-	-	-	-	0.0148(37)	-	-
*δ_K_* (kHz)	0.176(22)	-	-	-	-	-	-
κ ^[b]^	0.21	0.02	−0.28	0.36	−0.31	−0.01	−0.06
*a*/*b*/*c*^[c]^ (D)	y/y/y	n/n/y	y/y/y	n/n/y	y/n/y	n/n/y	n/n/y
*σ*^[d]^ (kHz)	4.4	5.4	6.7	3.5	6.2	4.9	5.4
N ^[e]^	85	15	49	21	43	19	19

^[a]^ *A*, *B* and *C* are the rotational constants. Δ*_J_*, Δ*_JK_*, Δ*_K_*, *δ_J_* and *δ_K_* are the centrifugal distortion constants. ^[b]^ Ray’s asymmetry parameter. ^[c]^ *a*, *b* and *c* are the type of transitions observed. ^[d]^ *σ* is the rms deviation of the fit. ^[e]^ N is the number of the fitted transitions. ^[f]^ Standard error in parentheses in the units of the last digit.

**Table 3:** Substitution and effective experimental bond lengths (Å), angles and dihedral angles (°) of conformer **I** of cyclododecanone and B3LYP-D3BJ/6-311++G(d,p) equilibrium structural parameters of all observed conformers of cyclododecanone.

Conformer	I	I	I	III	IV	V	VI	VII	II
Parameter	*r*_s_^[a]^	*r*_0_^[b]^	*r*_e_	*r*_e_	*r*_e_	*r*_e_	*r*_e_	*r*_e_	*r*_e_
*r*(C_2_-C_1_)	1.577(20)	1.528(15)	1.537	1.541	1.538	1.541	1.542	1.532	1.540
*r*(C_3_-C_2_)	1.486(14)	1.524(9)	1.537	1.540	1.538	1.542	1.530	1.532	1.537
*r*(C_4_-C_3_)	1.479(19)	1.527(16)	1.533	1.533	1.537	1.539	1.527	1.539	1.532
*r*(C_5_-C_4_)	1.579(28)	1.541(14)	1.535	1.536	1.536	1.532	1.527	1.539	1.536
*r*(C_6_-C_5_)	1.554(22)	1.523(12)	1.533	1.535	1.535	1.543	1.540	1.534	1.534
*r*(C_7_-C_6_)	1.437(24)	1.526(10) ^[c]^	1.525	1.525	1.525	1.520	1.529	1.526	1.524
*r*(C_12_-C_7_)	1.468(15)	1.526(10)	1.519	1.522	1.523	1.524	1.524	1.524	1.524
*r*(C_12_-C_11_)	1.541(9)	1.537(13)	1.544	1.541	1.532	1.536	1.534	1.534	1.538
*r*(C_11_-C_10_)	1.526(11)	1.533(14)	1.533	1.532	1.539	1.536	1.538	1.536	1.536
*r*(C_10_-C_9_)	1.533(10)	1.534(14)	1.537	1.531	1.541	1.532	1.534	1.538	1.531
*r*(C_9_-C_8_)	1.537(10)	1.527(10)	1.537	1.536	1.538	1.533	1.537	1.539	1.538
*r*(C_8_-C_1_)	1.522(10)	1.542(13)	1.541	1.540	1.533	1.535	1.542	1.540	1.540
∠(C_3_-C_2_-C_1_)	113.8(4)	114.3(2)	114.1	117.0	114.6	116.4	112.7	113.2	117.4
∠(C_4_-C_3_-C_2_)	116.8(17)	114.4(7)	113.9	114.3	113.9	117.1	116.3	114.7	114.0
∠(C_5_-C_4_-C_3_)	115.4(17)	114.5(8) ^[c]^	114.5	114.6	114.8	114.1	112.7	116.5	114.6
∠(C_6_-C_5_-C_4_)	113.0(4)	113.5(2)	113.9	114.6	113.5	113.4	114.0	116.4	113.6
∠(C_7_-C_6_-C_5_)	116.7(20)	114.2(8) ^[c]^	114.1	114.3	113.9	113.1	114.4	115.6	113.3
∠(C_12_-C_7_-C_6_)	125.2(27)	116.5(6) ^[c]^	116.8	117.6	117.2	117.2	117.0	116.1	118.2
∠(C_11_-C_12_-C_7_)	111.1(2)	111.5(2)	111.6	112.7	114.3	114.5	115.4	115.4	112.7
∠(C_12_-C_11_-C_10_)	112.7(5)	113.2(7)	113.5	113.0	116.2	114.1	114.7	114.2	114.1
∠(C_11_-C_10_-C_9_)	114.5(11)	113.8(10)	113.7	113.4	116.2	114.7	114.3	115.0	114.6
∠(C_10_-C_9_-C_8_)	113.8(3)	114.1(2)	114.1	114.0	115.9	113.1	115.1	114.1	114.2
∠(C_9_-C_8_-C_1_)	113.2(9)	114.1(8)	114.1	116.7	114.1	114.3	117.4	114.4	117.5
∠(C_8_-C_1_-C_2_)	111.2(14)	114.8(7)	113.9	116.5	114.2	116.6	116.1	114.6	116.7
τ(C_4_-C_3_-C_2_-C_1_)	−63.0(21)	−68.1(7) ^[c]^	−68.1	−64.3	−61.7	84.0	177.1	172.8	−62.2
τ(C_5_-C_4_-C_3_-C_2_)	170.8(2)	171.4(3)	172.8	178.3	156.9	64.4	−176.4	−84.6	175.9
τ(C_6_-C_5_-C_4_-C_3_)	−66.0(23)	−68.6(10)	−66.1	−61.5	−64.4	−178.3	64.1	68.0	−64.4
τ(C_7_-C_6_-C_5_-C_4_)	−66.0(24)	−63.8(10) ^[c]^	−65.1	−63.5	−59.3	60.4	−75.2	−87.2	−62.7
τ(C_12_-C_7_-C_6_-C_5_)	152.0(9)	153.8(3) ^[c]^	151.8	150.6	152.5	70.2	134.3	162.4	146.2
τ(C_11_-C_12_-C_7_-C_6_)	−71.1(13)	−75.0(5) ^[c]^	−75.2	−62.7	−159.8	−149.5	−159.6	−164.9	−138.1
τ(C_10_-C_11_-C_12_-C_7_)	−70.6(16)	−65.1(6) ^[c]^	−65.1	−55.1	62.3	56.3	67.1	59.2	57.3
τ(C_12_-C_11_-C_10_-C_9_)	170.1(2)	170.1(2)	171.5	164.0	63.8	56.5	60.6	61.3	58.2
τ(C_11_-C_10_-C_9_-C_8_)	−68.1(13)	−69.1(4)	−68.3	−166.6	−86.5	−165.5	−173.8	−155.3	179.3
τ(C_10_-C_9_-C_8_-C_1_)	−70.3(14)	−68.8(8) ^[c]^	−68.8	56.2	−68.9	164.6	62.3	55.1	62.9
τ(C_9_-C_8_-C_1_-C_2_)	150.2(4)	149.5(4)	148.0	59.5	173.2	−56.7	54.4	51.5	78.4
τ(C_8_-C_1_-C_2_-C_3_)	−74.0(21)	−68.2(3)	−69.0	−83.6	−59.6	−58.3	−85.9	−168.8	−72.4

^[a]^ The substitution structure has been determined from the atomic coordinates including Costain’s error and with signs taken from the B3LYP-D3BJ calculated structure. ^[b]^ Effective structure; non-fitted parameters were fixed to the B3LYP-D3BJ/6-311++G(d,p) values. ^[c]^ Derived from the determined *r*_0_ structure, not fitted directly.

## Data Availability

The data presented in this study are available in the Supplementary Material.

## Supplementary Materials

The following are available online. Table S1: Ab initio spectroscopic parameters for the conformers of cyclododecanone, Tables S2–S20: Measured frequencies of observed species, Table S21: Benchmarking of the theoretical rotational constants against the experimental (A_calc_ − A_exp_)/A_exp_ × 100%, Table S22: Experimental spectroscopic parameters for all singly substituted ^13^C isotopic species of conformer **I** of cyclododecanone, Table S23: Substitution coordinates of the heavy atoms of conformer **I** of cyclododecanone in Å, Tables S24–S30: Cartesian coordinates of observed species.

## Sample Availability

Samples of the compounds are commercially available.
