# Supplementary material for: Seven Conformations of the Macrocycle Cyclododecanone Unveiled by Microwave Spectroscopy
Source: Molecules. 2021 Aug 26;26(17):5162. doi: 10.3390/molecules26175162 (PMC8433831; doi:10.3390/molecules26175162)
Supplement: Supplementary file 1 [file molecules-26-05162-s001.zip › molecules-1320281-supplementary.pdf]

Seven conformations of the macrocycle cyclododecanone unveiled by microwave spectroscopy

Ecaterina Burevschi, M. Eugenia Sanz*

Department of Chemistry
King's College London
London SE1 1DB, United Kingdom

Supplementary Information

Table of Contents

Table S1. *Ab initio* spectroscopic parameters for the conformers of cyclododecanone within 1000 cm^{-1}.

Table S2. Measured frequencies and residuals (in MHz) for the rotational transitions of the parent species of conformer **I**.

Table S3. Measured frequencies and residuals (in MHz) for the rotational transitions of the $^{13}C_1$ isotopologue of conformer **I**.

Table S4. Measured frequencies and residuals (in MHz) for the rotational transitions of the $^{13}C_2$ isotopologue of conformer **I**.

Table S5. Measured frequencies and residuals (in MHz) for the rotational transitions of the $^{13}C_3$ isotopologue of conformer **I**.

Table S6. Measured frequencies and residuals (in MHz) for the rotational transitions of the $^{13}C_4$ isotopologue of conformer **I**.

Table S7. Measured frequencies and residuals (in MHz) for the rotational transitions of the $^{13}C_5$ isotopologue of conformer **I**.

Table S8. Measured frequencies and residuals (in MHz) for the rotational transitions of the $^{13}C_6$ isotopologue of conformer **I**.

Table S9. Measured frequencies and residuals (in MHz) for the rotational transitions of the $^{13}C_7$ isotopologue of conformer **I**.

Table S10. Measured frequencies and residuals (in MHz) for the rotational transitions of the $^{13}C_8$ isotopologue of conformer **I**.

Table S11. Measured frequencies and residuals (in MHz) for the rotational transitions of the $^{13}C_9$ isotopologue of conformer **I**.

Table S12. Measured frequencies and residuals (in MHz) for the rotational transitions of the $^{13}C_{10}$ isotopologue of conformer **I**.

Table S13. Measured frequencies and residuals (in MHz) for the rotational transitions of the $^{13}C_{11}$ isotopologue of conformer **I**.

Table S14. Measured frequencies and residuals (in MHz) for the rotational transitions of the $^{13}C_{12}$ isotopologue of conformer **I**.

Table S15. Measured frequencies and residuals (in MHz) for the rotational transitions of conformer **II**.

Table S16. Measured frequencies and residuals (in MHz) for the rotational transitions of the parent species of conformer **III**.

Table S17. Measured frequencies and residuals (in MHz) for the rotational transitions of conformer **IV**.

Table S18. Measured frequencies and residuals (in MHz) for the rotational transitions of conformer **V**.

Table S19. Measured frequencies and residuals (in MHz) for the rotational transitions of conformer **VI**.

Table S20. Measured frequencies and residuals (in MHz) for the rotational transitions of conformer **VII**.

Table S21. Benchmarking of the theoretical rotational constants against the experimental $(A_{calc} - A_{exp})/A_{exp} \times 100\%$.

Table S22. Experimental spectroscopic parameters for all singly substituted ^{13}C isotopic species of conformer **I** of cyclododecanone.

Table S23. Substitution coordinates of the heavy atoms of conformer **I** of cyclododecanone in Å.

Table S24. The Cartesian coordinates of the conformer **I** of cyclododecanone from B3LYP-D3BJ/3-611++G(d,p).

Table S25. The Cartesian coordinates of the conformer **II** of cyclododecanone from B3LYP-D3BJ/3-611++G(d,p).

Table S26. The Cartesian coordinates of the conformer **III** of cyclododecanone from B3LYP-D3BJ/3-611++G(d,p).

Table S27. The Cartesian coordinates of the conformer **IV** of cyclododecanone from B3LYP-D3BJ/3-611++G(d,p).

Table S28. The Cartesian coordinates of the conformer **V** of cyclododecanone from B3LYP-D3BJ/3-611++G(d,p).

Table S29. The Cartesian coordinates of the conformer **VI** of cyclododecanone from B3LYP-D3BJ/3-611++G(d,p).

Table S30. The Cartesian coordinates of the conformer **VII** of cyclododecanone from B3LYP-D3BJ/3-611++G(d,p).

Table S1. *Ab initio*[a] spectroscopic parameters for the conformers of cyclododecanone within 1000 cm^{-1}.

	**Conformer I**		**Conformer II**		**Conformer III**		**Conformer IV**	
	B3LYP	MP2	B3LYP	MP2	B3LYP	MP2	B3LYP	MP2
A^{b} (MHz)	872.3	888.5	896.1	918.6	967.3	979.6	858.0	880.7
B (MHz)	691.1	693.2	693.2	697.0	632.2	641.2	740.9	741.3
C (MHz)	447.0	453.0	450.7	459.1	450.4	459.4	461.2	468.1
μ_a (D)	0.9	0.8	0.8	0.6	0.9	0.7	-0.2	-0.2
μ_b (D)	0.2	0.2	0.2	0.4	-0.6	-0.5	0.5	0.5
μ_c (D)	-2.6	-2.4	-2.3	-2.2	2.6	2.4	2.4	2.2
ΔE^{c} (cm^{-1})	0.0	0.0	469.1	516.4	461.6	401.9	583.8	574.0
ΔE_0^{d} (cm^{-1})	**0.0**	**0.0**	**446.4**	**527.8**	**455.8**	**443.3**	**575.0**	**585.1**
ΔG^{e} (cm^{-1})	0.0	0.0	269.7	566.5	537.7	579.2	521.7	649.4

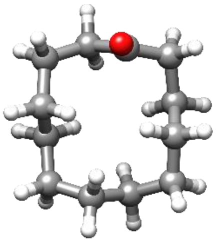
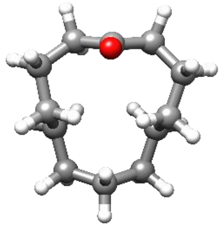
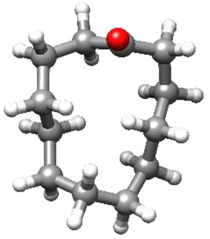
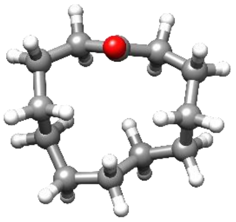

	**Conformer V**		**Conformer VI**		**Conformer VII**		**Conformer VIII**	
	B3LYP	MP2	B3LYP	MP2	B3LYP	MP2	B3LYP	MP2
A^{b} (MHz)	988.5	1002.0	914.3	925.0	943.7	951.8	890.5	915.4
B (MHz)	630.1	635.2	673.7	680.3	682.2	687.6	678.6	675.0
C (MHz)	445.6	451.7	442.3	447.9	453.3	458.3	451.7	455.8
μ_a (D)	1.3	1.1	0.3	0.2	-0.1	-0.1	0.5	0.6
μ_b (D)	0.4	0.4	-0.3	-0.3	-0.3	-0.2	-1.1	-1.0
μ_c (D)	-2.5	-2.3	-2.4	-2.2	2.4	2.2	2.6	2.4
ΔE^{c} (cm^{-1})	495.3	452.6	645.6	604.9	698.0	726.3	823.8	962.2
ΔE_0^{d} (cm^{-1})	**590.6**	**508.3**	**592.1**	**572.0**	**611.2**	**673.8**	**780.7**	**956.5**
ΔG^{e} (cm^{-1})	780.9	663.3	583.4	538.2	594.6	696.4	595.2	875.0

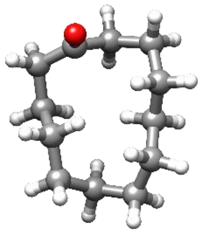
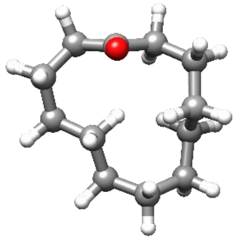
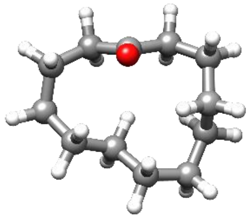
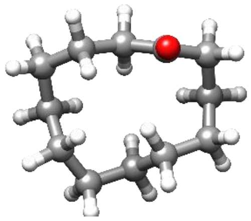

	**Conformer IX**		**Conformer X**		**Conformer XI (HP)**		**Conformer XII**	
	B3LYP	MP2	B3LYP	MP2	B3LYP	MP2	B3LYP	MP2
A^{b} (MHz)	944.1	956.8	849.8	855.0	910.0	924.9	887.4	898.3
B (MHz)	699.1	699.7	746.4	753.1	694.6	699.7	700.6	706.9
C (MHz)	477.3	483.3	480.6	487.0	468.6	475.5	462.9	470.0
μ_a (D)	0.2	0.3	-0.1	-0.1	-0.6	-0.6	0.3	0.3
μ_b (D)	-1.1	-0.9	0.7	0.7	0.2	0.2	0.2	0.2
μ_c (D)	-2.1	-2.0	-2.5	-2.3	2.6	2.4	-2.6	-2.4
ΔE^{c} (cm^{-1})	851.1	1031.2	824.8	918.9	845.7	848.8	860.3	986.5
ΔE_0^{d} (cm^{-1})	**802.8**	**1005.2**	**816.2**	**960.4**	**843.0**	**879.9**	**858.4**	**1030.2**
ΔG^{e} (cm^{-1})	802.8	1123.9	843.4	1003.0	848.0	885.8	894.6	1126.1

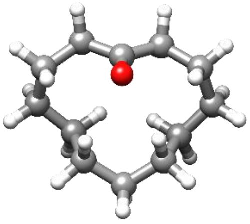 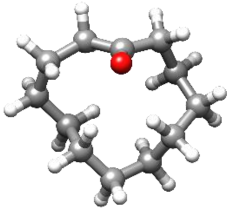 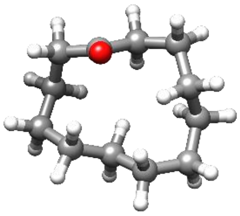 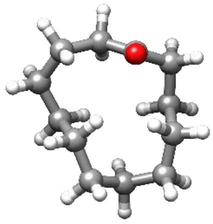

	Conformer XIII		Conformer XIV		Conformer XV		Conformer XVI	
	B3LYP	MP2	B3LYP	MP2	B3LYP	MP2	B3LYP	MP2
A^b (MHz)	945.6	946.6	898.8	908.9	914.8	920.7	881.9	889.6
B (MHz)	687.7	696.6	661.4	665.8	663.7	674.4	686.0	694.1
C (MHz)	459.7	465.3	437.4	442.1	442.6	449.5	460.5	466.3
μ_a (D)	-0.5	-0.5	0.5	0.5	-0.6	-0.4	0.8	0.6
μ_b (D)	0.5	0.4	-0.9	-0.7	0.3	0.3	0.8	0.8
μ_c (D)	-2.3	-2.1	2.5	2.3	2.6	2.4	2.4	2.3
ΔE^c (cm^{-1})	897.4	1041.0	916.8	1011.4	870.4	752.2	958.1	978.3
ΔE_0^d (cm^{-1})	**867.1**	**1003.7**	**893.0**	**976.2**	**898.7**	**757.2**	**921.8**	**944.4**
ΔG^e (cm^{-1})	932.5	1020.8	796.7	788.6	1031.5	860.1	870.7	878.1

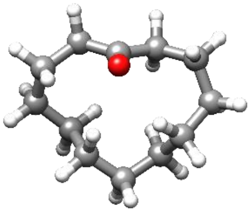 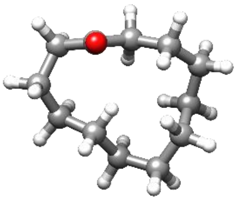 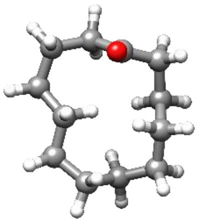 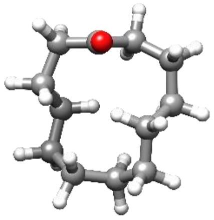

	Conformer XVII		Conformer XVIII	
	B3LYP	MP2	B3LYP	MP2
A^b (MHz)	862.1	878.8	948.9	959.4
B (MHz)	687.6	693.5	636.2	643.4
C (MHz)	442.6	452.4	451.9	458.5
μ_a (D)	-0.7	-0.4	0.8	0.7
μ_b (D)	-1.6	-1.4	-0.9	-0.8
μ_c (D)	2.4	2.3	2.7	2.5
ΔE^c (cm^{-1})	981.3	1099.9	919.0	824.0
ΔE_0^d (cm^{-1})	**958.4**	**1094.1**	**1008.0**	**922.5**
ΔG^e (cm^{-1})	870.7	960.6	1108.8	1021.7

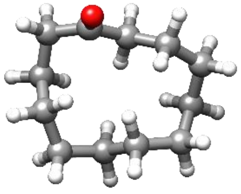 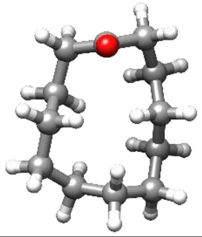

[a] B3LYP-D3BJ and MP2 with 6-311++G(d,p) basis set. [b] A, B, C are the rotational constants; μ_a, μ_b and μ_c are the electric dipole moment components. [c] Relative electronic energies. [d] Relative electronic energies including the zero-point correction. [e] Gibbs free energies at 417 K.

Table S2. Measured frequencies and residuals (in MHz) for the rotational transitions of the parent species of conformer **I**.

J'	K'_{-1}	K'_{+1}	J"	$K"_{-1}$	$K"_{+1}$	ν_{obs}	$\nu_{obs}-\nu_{calc}$
2	1	2	1	1	1	2047.9862	-0.0014
2	0	2	1	0	1	2154.4526	-0.0069
2	1	1	1	1	0	2550.6065	0.0067
2	1	1	1	0	1	2967.0800	-0.0027
3	0	3	2	1	2	2982.8671	0.0044
3	1	3	2	1	2	2996.9284	-0.0007
3	0	3	2	0	2	3041.5672	0.0003
3	1	3	2	0	2	3055.6345	0.0013
2	2	0	1	1	0	3190.9575	-0.0041
2	2	1	1	1	1	3297.4298	-0.0050
3	1	2	2	1	1	3687.4884	0.0000
3	2	1	2	2	0	3856.3025	-0.0048
4	0	4	3	1	3	3909.3566	-0.0036
4	1	4	3	1	3	3912.1279	0.0000
4	0	4	3	0	3	3923.4270	0.0004
4	1	4	3	0	3	3926.1921	-0.0022
4	1	3	3	2	2	4385.8828	0.0111
4	2	3	3	2	2	4476.1959	-0.0022
3	2	1	2	1	1	4496.6700	0.0010
4	1	3	3	1	2	4642.8506	0.0003
3	2	2	2	1	2	4698.3986	0.0127
4	2	3	3	1	2	4733.1649	-0.0117
4	3	2	3	3	1	4782.4227	0.0006
5	0	5	4	1	4	4814.0629	0.0108
5	1	5	4	1	4	4814.5413	-0.0013
5	0	5	4	0	4	4816.8190	-0.0008
5	1	5	4	0	4	4817.3097	-0.0006
3	3	1	2	2	0	4833.3003	0.0031
3	3	0	2	2	0	4896.6443	-0.0079
3	3	1	2	2	1	4978.1331	0.0023
3	3	0	2	2	1	5041.4823	-0.0036
4	3	1	3	3	0	5056.9071	-0.0045
4	2	2	3	2	1	5128.4440	-0.0024
5	1	4	4	2	3	5412.5518	0.0034
5	2	4	4	2	3	5436.0977	-0.0035
5	1	4	4	1	3	5502.8757	0.0010
5	2	4	4	1	3	5526.4325	0.0050
6	1	6	5	1	5	5713.6228	-0.0001
6	0	6	5	0	5	5714.0253	-0.0066
4	3	2	3	2	1	5759.4119	-0.0002
5	3	3	4	3	2	5896.3194	-0.0043
4	2	2	3	1	2	5937.6256	-0.0014
5	4	2	4	4	1	6060.3312	-0.0005
4	3	1	3	2	1	6097.2585	0.0020
4	1	3	3	0	3	6101.3953	0.0003
4	2	3	3	1	3	6177.6549	0.0000
5	4	1	4	4	0	6205.3253	-0.0023
5	2	3	4	2	2	6222.4889	0.0011
4	3	2	3	2	2	6311.6153	0.0011
6	1	5	5	2	4	6350.6128	-0.0039
6	2	5	5	2	4	6355.7823	-0.0029
6	1	5	5	1	4	6374.1669	-0.0026
6	2	5	5	1	4	6379.3422	0.0042
5	3	2	4	3	1	6457.0938	-0.0023
5	3	3	4	2	2	6527.2989	0.0095
4	4	1	3	3	0	6618.3448	-0.0010
4	4	0	3	3	0	6641.2434	0.0001
4	3	1	3	2	2	6649.4600	0.0013
4	4	1	3	3	1	6681.6995	-0.0013
4	4	0	3	3	1	6704.6007	0.0024
6	2	4	5	3	3	6819.7880	0.0094
6	3	4	5	3	3	6925.7809	-0.0020
6	2	4	5	2	3	7124.5692	-0.0110
6	3	4	5	2	3	7230.5949	0.0104
6	4	3	5	4	2	7252.3311	0.0064
7	2	6	6	2	5	7259.4120	0.0027

7	1	6	6	1	5	7263.5559	-0.0003
7	2	6	6	1	5	7264.5808	0.0030
6	5	2	5	5	1	7300.2319	0.0009
5	3	2	4	2	2	7425.9071	0.0009
8	0	8	7	1	7	7510.3538	0.0013
8	1	8	7	1	7	7510.3538	0.0013
8	0	8	7	0	7	7510.3538	0.0013
8	1	8	7	0	7	7510.3538	0.0013
5	2	3	4	1	3	7517.2633	-0.0013
6	4	2	5	4	1	7665.0885	-0.0006
5	1	4	4	0	4	7680.8438	0.0007
5	2	4	4	1	4	7701.6282	0.0001
6	3	3	5	3	2	7710.7378	0.0004
5	3	3	4	2	3	7731.7384	-0.0015
5	4	1	4	3	1	7789.6585	-0.0008
7	3	5	6	3	4	7885.0229	-0.0009
5	4	2	4	3	2	7959.6108	0.0005
7	2	5	6	2	4	7962.1564	-0.0045
7	3	5	6	2	4	7991.0215	-0.0066

Table S3. Measured frequencies and residuals (in MHz) for the rotational transitions of the $^{13}C_1$ isotopologue of conformer **I**.

J'	K'_{-1}	K'_{+1}	J"	$K"_{-1}$	$K"_{+1}$	ν_{obs}	ν_{obs}-ν_{calc}
3	2	1	2	1	1	4470.5727	0.0050
3	3	0	2	2	0	4886.8864	-0.0032
3	3	1	2	2	1	4967.1840	0.0017
4	2	2	3	1	2	5894.6243	0.0051
4	3	1	3	2	1	6070.4794	0.0078
4	3	2	3	2	2	6286.4521	0.0055
4	4	0	3	3	0	6630.9947	-0.0023
4	4	1	3	3	1	6669.4630	0.0014
5	3	2	4	2	2	7378.4794	-0.0066
5	2	3	4	1	3	7459.4194	-0.0042
5	4	1	4	3	1	7766.6218	-0.0051
5	4	2	4	3	2	7932.7193	-0.0002

Table S4. Measured frequencies and residuals (in MHz) for the rotational transitions of the $^{13}C_2$ isotopologue of conformer **I**.

J'	K'_{-1}	K'_{+1}	J"	$K"_{-1}$	$K"_{+1}$	ν_{obs}	ν_{obs}-ν_{calc}
3	2	1	2	1	1	4467.0738	-0.0035
3	3	0	2	2	0	4869.4408	-0.0029
3	3	1	2	2	1	4950.3413	0.0023
4	3	1	3	2	1	6059.2971	-0.0049
4	1	3	3	0	3	6060.0375	0.0036
4	2	3	3	1	3	6137.8253	0.0136
4	4	0	3	3	0	6605.2172	0.0015
4	4	1	3	3	1	6645.0397	0.0008
5	3	2	4	2	2	7375.8864	0.0016
5	2	3	4	1	3	7464.4933	-0.0046
5	3	3	4	2	3	7682.4172	-0.0017
5	4	1	4	3	1	7744.0833	0.0065
5	4	2	4	3	2	7912.4822	-0.0110

Table S5. Measured frequencies and residuals (in MHz) for the rotational transitions of the $^{13}C_3$ isotopologue of conformer **I**.

J'	K'_{-1}	K'_{+1}	J"	$K"_{-1}$	$K"_{+1}$	ν_{obs}	ν_{obs}-ν_{calc}
2	2	0	1	1	0	3172.4206	-0.0009
3	2	1	2	1	1	4478.8613	-0.0097
3	3	0	2	2	0	4863.9617	0.0079
3	3	1	2	2	1	4945.5705	-0.0029
4	2	2	3	1	2	5920.1820	-0.0069
4	3	1	3	2	1	6067.1539	0.0047
4	1	3	3	0	3	6081.0965	0.0008
4	2	3	3	1	3	6151.4691	0.0057
4	3	2	3	2	2	6278.4075	0.0078
4	4	1	3	3	1	6636.2535	-0.0048
5	3	2	4	2	2	7399.7829	0.0047
5	3	3	4	2	3	7697.4208	-0.0012
5	4	1	4	3	1	7743.1686	-0.0042
5	4	2	4	3	2	7914.3196	-0.0020

Table S6. Measured frequencies and residuals (in MHz) for the rotational transitions of the $^{13}C_4$ isotopologue of conformer **I**.

J’	K'_{-1}	K'_{+1}	J”	K''_{-1}	K''_{+1}	ν_{obs}	$\nu_{obs}-\nu_{calc}$
3	2	1	2	1	1	4482.0418	0.0022
3	3	0	2	2	0	4863.6382	0.0095
3	3	1	2	2	1	4945.4462	0.0058
4	2	2	3	1	2	5926.0976	-0.0034
4	3	1	3	2	1	6069.7769	0.0073
4	2	3	3	1	3	6155.3042	-0.0086
4	3	2	3	2	2	6280.5171	0.0039
4	4	0	3	3	0	6593.5687	-0.0012
4	4	1	3	3	1	6635.5846	-0.0009
5	3	2	4	2	2	7405.9726	0.0042
5	2	4	4	1	4	7675.9080	0.0035
5	4	1	4	3	1	7744.2274	-0.0063
5	4	2	4	3	2	7916.0184	-0.0094

Table S7. Measured frequencies and residuals (in MHz) for the rotational transitions of the $^{13}C_5$ isotopologue of conformer **I**.

J’	K'_{-1}	K'_{+1}	J”	K''_{-1}	K''_{+1}	ν_{obs}	$\nu_{obs}-\nu_{calc}$
3	2	1	2	1	1	4473.0510	0.0115
3	3	0	2	2	0	4862.2706	-0.0002
3	3	1	2	2	1	4943.7396	0.0018
4	2	2	3	1	2	5910.5047	-0.0067
4	3	1	3	2	1	6061.2276	-0.0022
4	3	2	3	2	2	6273.1947	0.0103
4	4	0	3	3	0	6593.2107	0.0004
5	3	2	4	2	2	7389.0269	0.0054
5	3	3	4	2	3	7688.8439	-0.0087
5	4	1	4	3	1	7738.3317	-0.0045
5	4	2	4	3	2	7908.8884	-0.0015

Table S8. Measured frequencies and residuals (in MHz) for the rotational transitions of the $^{13}C_6$ isotopologue of conformer **I**.

J’	K'_{-1}	K'_{+1}	J”	K''_{-1}	K''_{+1}	ν_{obs}	$\nu_{obs}-\nu_{calc}$
2	2	0	1	1	0	3179.9957	-0.0106
3	2	1	2	1	1	4474.9071	-0.0068
3	3	1	2	2	1	4963.7167	0.0064
4	2	2	3	1	2	5904.1845	-0.0020
4	1	3	3	0	3	6068.5634	0.0071
4	3	1	3	2	1	6072.4505	0.0016
4	2	3	3	1	3	6148.8934	-0.0066
4	3	2	3	2	2	6287.2536	0.0022
4	4	0	3	3	0	6624.4403	-0.0013
4	4	1	3	3	1	6663.6852	0.0014
5	3	2	4	2	2	7387.5969	0.0108
5	2	3	4	1	3	7473.0248	-0.0058
5	3	3	4	2	3	7697.0055	0.0004
5	4	1	4	3	1	7764.0896	-0.0027

Table S9. Measured frequencies and residuals (in MHz) for the rotational transitions of the $^{13}C_7$ isotopologue of conformer **I**.

J'	K'_{-1}	K'_{+1}	J"	$K"_{-1}$	$K"_{+1}$	ν_{obs}	ν_{obs}-ν_{calc}
3	2	1	2	1	1	4479.6446	0.0091
3	3	0	2	2	0	4891.5926	0.0070
3	3	1	2	2	1	4972.3154	0.0035
4	2	2	3	1	2	5909.0484	-0.0036
4	3	1	3	2	1	6080.2986	0.0032
4	2	3	3	1	3	6155.9684	-0.0006
4	3	2	3	2	2	6296.0461	-0.0001
4	4	1	3	3	1	6675.6513	-0.0038
5	3	2	4	2	2	7394.6437	0.0003
5	3	3	4	2	3	7706.2133	-0.0019
5	4	1	4	3	1	7776.0255	-0.0063

Table S10. Measured frequencies and residuals (in MHz) for the rotational transitions of the $^{13}C_8$ isotopologue of conformer **I**.

J'	K'_{-1}	K'_{+1}	J"	$K"_{-1}$	$K"_{+1}$	ν_{obs}	ν_{obs}-ν_{calc}
3	2	1	2	1	1	4470.7763	0.0030
3	3	0	2	2	0	4885.8461	0.0065
3	3	1	2	2	1	4966.2500	0.0023
4	2	2	3	1	2	5895.5499	-0.0042
4	1	3	3	0	3	6060.8839	0.0086
4	3	1	3	2	1	6070.0972	-0.0037
4	3	2	3	2	2	6285.9983	-0.0007
4	4	0	3	3	0	6629.3987	0.0000
4	4	1	3	3	1	6668.0277	-0.0033
5	3	2	4	2	2	7379.1316	0.0077
5	2	3	4	1	3	7460.9559	-0.0078
5	4	1	4	3	1	7765.3418	-0.0043

Table S11. Measured frequencies and residuals (in MHz) for the rotational transitions of the $^{13}C_9$ isotopologue of conformer **I**.

J'	K'_{-1}	K'_{+1}	J"	$K"_{-1}$	$K"_{+1}$	ν_{obs}	ν_{obs}-ν_{calc}
2	2	0	1	1	0	3169.6056	-0.0115
3	2	1	2	1	1	4468.9290	0.0064
3	2	2	2	1	2	4668.2718	-0.0128
3	3	0	2	2	0	4862.6614	-0.0029
3	3	1	2	2	1	4943.9062	0.0042
4	2	2	3	1	2	5902.8770	0.0024
4	3	1	3	2	1	6057.8209	-0.0017
4	3	2	3	2	2	6270.4340	0.0025
4	4	0	3	3	0	6594.5753	0.0063
4	4	1	3	3	1	6635.2487	0.0016
5	3	3	4	2	3	7683.1577	0.0034
5	4	1	4	3	1	7736.9114	0.0022
5	4	2	4	3	2	7906.6513	-0.0068

Table S12. Measured frequencies and residuals (in MHz) for the rotational transitions of the $^{13}C_{10}$ isotopologue of conformer **I**.

J'	K'_{-1}	K'_{+1}	J"	$K"_{-1}$	$K"_{+1}$	ν_{obs}	ν_{obs}-ν_{calc}
2	2	0	1	1	0	3173.2110	-0.0042
3	2	1	2	1	1	4481.6301	0.0030
3	3	0	2	2	0	4864.3305	0.0007
3	3	1	2	2	1	4946.1007	0.0035
4	2	2	3	1	2	5925.0395	0.0045
4	3	1	3	2	1	6069.7076	-0.0091
4	2	3	3	1	3	6154.9048	-0.0011
4	3	2	3	2	2	6280.6435	-0.0050
5	3	2	4	2	2	7404.9953	-0.0001
5	4	1	4	3	1	7744.8660	0.0060

Table S13. Measured frequencies and residuals (in MHz) for the rotational transitions of the $^{13}C_{11}$ isotopologue of conformer **I**.

J'	K'_{-1}	K'_{+1}	J"	$K"_{-1}$	$K"_{+1}$	ν_{obs}	ν_{obs}-ν_{calc}
3	3	0	2	2	0	4862.8183	0.0082
3	3	1	2	2	1	4944.4437	0.0012
4	2	2	3	1	2	5922.2546	0.0023
4	3	1	3	2	1	6067.4833	-0.0088
4	1	3	3	0	3	6082.7368	-0.0013
4	2	3	3	1	3	6152.3226	0.0074
4	3	2	3	2	2	6278.2861	-0.0098
4	4	0	3	3	0	6592.7185	0.0048
4	4	1	3	3	1	6634.4679	-0.0042
5	3	2	4	2	2	7401.7854	0.0038
5	3	3	4	2	3	7698.2759	-0.0019
5	4	1	4	3	1	7742.3892	0.0029
5	4	2	4	3	2	7913.6761	-0.0027

Table S14. Measured frequencies and residuals (in MHz) for the rotational transitions of the $^{13}C_{12}$ isotopologue of conformer **I**.

J'	K'_{-1}	K'_{+1}	J"	$K"_{-1}$	$K"_{+1}$	ν_{obs}	ν_{obs}-ν_{calc}
2	2	0	1	1	0	3173.6023	-0.0040
3	2	1	2	1	1	4472.3197	-0.0011
3	3	0	2	2	0	4869.9388	-0.0017
3	3	1	2	2	1	4951.0496	-0.0013
4	2	2	3	1	2	5905.6526	0.0007
4	3	1	3	2	1	6064.1002	0.0031
4	1	3	3	0	3	6068.5634	-0.0089
4	3	2	3	2	2	6277.3674	0.0032
4	4	0	3	3	0	6605.0041	-0.0035
4	4	1	3	3	1	6645.3194	0.0002
5	3	2	4	2	2	7385.7537	-0.0091
5	2	3	4	1	3	7476.9315	0.0040
5	3	3	4	2	3	7689.9191	0.0122
5	4	1	4	3	1	7747.1270	0.0017
5	4	2	4	3	2	7916.3410	-0.0006

Table S15. Measured frequencies and residuals (in MHz) for the rotational transitions of conformer **II**.

J'	K'_{-1}	K'_{+1}	J"	$K"_{-1}$	$K"_{+1}$	ν_{obs}	ν_{obs}-ν_{calc}
4	1	3	3	0	3	5996.7302	-0.0032
5	1	4	4	0	4	7588.5744	0.0109
3	2	1	2	1	1	4474.7078	0.0037
4	2	2	3	1	2	5834.9445	-0.0058
5	2	3	4	1	3	7342.0322	-0.0010
4	2	3	3	1	3	6153.2563	-0.0069
3	3	0	2	2	0	5021.9413	0.0120
4	3	1	3	2	1	6146.8083	0.0003
5	3	2	4	2	2	7364.0171	0.0017
3	3	1	2	2	1	5093.0474	0.0014
4	3	2	3	2	2	6368.4064	0.0011
5	3	3	4	2	3	7726.2372	-0.0020
4	4	0	3	3	0	6829.9276	-0.0004
4	4	1	3	3	1	6856.3795	-0.0079
5	4	1	4	3	1	7939.6690	-0.0017

Table S16. Measured frequencies and residuals (in MHz) for the rotational transitions of the parent species of conformer **III**.

J'	K'_{-1}	K'_{+1}	J"	$K"_{-1}$	$K"_{+1}$	ν_{obs}	ν_{obs}-ν_{calc}
3	0	3	2	1	2	2871.2892	0.0151
2	1	1	1	0	1	2881.7420	-0.0021
3	1	3	2	1	2	2968.0578	-0.0044
3	0	3	2	0	2	3073.0396	0.0050
2	2	0	1	1	0	3411.0624	-0.0013
3	1	2	2	1	1	3509.4802	-0.0062
2	2	1	1	1	1	3536.8548	-0.0073
4	0	4	3	1	3	3872.5035	-0.0052
4	1	4	3	1	3	3911.1105	0.0018
4	0	4	3	0	3	3969.2944	-0.0025
3	1	2	2	0	2	4265.1235	0.0005
4	2	3	3	2	2	4322.9323	-0.0109
3	2	1	2	1	1	4523.2695	0.0005
4	1	3	3	1	2	4580.5476	-0.0037
3	2	2	2	1	2	4813.9433	-0.0096
5	0	5	4	1	4	4822.4448	-0.0013
5	1	5	4	1	4	4836.2002	0.0070
5	1	5	4	0	4	4874.7855	-0.0077
5	2	4	4	2	3	5332.7728	0.0099
3	3	1	2	2	0	5350.3424	0.0115
3	3	0	2	2	0	5362.7865	0.0037
3	3	1	2	2	1	5409.1642	0.0064
5	1	4	4	1	3	5555.6809	0.0020
4	2	2	3	1	2	5735.1231	-0.0020
6	0	6	5	1	5	5746.6377	-0.0021
6	1	6	5	1	5	5751.2032	-0.0034
6	0	6	5	0	5	5760.3900	0.0031
4	1	3	3	0	3	5772.6360	-0.0037
5	2	3	4	2	2	5917.5364	0.0045
4	2	3	3	1	3	6168.8366	0.0027
6	1	5	5	2	4	6182.2575	0.0155
6	2	5	5	2	4	6306.9305	0.0049
4	3	1	3	2	1	6409.4431	-0.0004
6	1	5	5	1	4	6452.3062	-0.0022
6	2	5	5	1	4	6576.9814	-0.0106
4	3	2	3	2	2	6591.1270	0.0021
7	0	7	6	1	6	6660.1861	0.0075
7	1	7	6	1	6	6661.6125	-0.0144
7	0	7	6	0	6	6664.7527	0.0074
5	2	3	4	1	3	7072.0972	-0.0084
4	4	1	3	3	0	7311.0447	0.0114
4	4	0	3	3	0	7313.2001	-0.0003
4	4	1	3	3	1	7323.4857	0.0005
4	4	0	3	3	1	7325.6459	-0.0064
7	1	6	6	1	5	7325.8971	-0.0024
5	1	4	4	0	4	7359.0263	0.0046
5	3	2	4	2	2	7460.6345	-0.0046
5	2	4	4	1	4	7590.4880	-0.0001
5	3	3	4	2	3	7837.4134	-0.0053

Table S17. Measured frequencies and residuals (in MHz) for the rotational transitions of conformer **IV**.

J'	K'_{-1}	K'_{+1}	J''	K''_{-1}	K''_{+1}	ν_{obs}	ν_{obs}-ν_{calc}
2	1	1	1	0	1	3088.1067	0.0034
2	2	0	1	1	0	3252.4771	-0.0059
2	2	1	1	1	1	3345.6626	-0.0059
3	2	1	2	1	1	4658.4603	0.0025
3	1	2	2	0	2	4697.8200	0.0056
3	2	2	2	1	2	4825.3180	-0.0073
3	3	0	2	2	0	4952.8270	0.0007
3	3	1	2	2	1	5038.3542	-0.0023
4	2	2	3	1	2	6203.2278	-0.0039
4	3	1	3	2	1	6268.6642	-0.0027
4	1	3	3	0	3	6341.2806	-0.0024
4	2	3	3	1	3	6377.7866	0.0057
4	3	2	3	2	2	6461.9631	-0.0050
4	4	0	3	3	0	6693.4340	0.0045
4	4	1	3	3	1	6747.2680	-0.0013
5	3	2	4	2	2	7727.3472	-0.0133
5	2	3	4	1	3	7859.1029	-0.0117
5	4	1	4	3	1	7937.9156	-0.0041
5	1	4	4	0	4	7956.2172	0.0134
5	2	4	4	1	4	7963.6123	0.0007
5	3	3	4	2	3	7971.6436	-0.0095

Table S18. Measured frequencies and residuals (in MHz) for the rotational transitions of conformer **V**.

J'	K'_{-1}	K'_{+1}	J"	$K"_{-1}$	$K"_{+1}$	ν_{obs}	ν_{obs}-ν_{calc}
2	1	1	1	0	1	2890.2417	-0.0115
3	1	3	2	1	2	2934.5141	-0.0033
3	0	3	2	0	2	3046.6988	-0.0013
2	2	0	1	1	0	3467.0124	0.0029
3	1	2	2	1	1	3482.8249	-0.0042
2	2	1	1	1	1	3596.9191	0.0007
4	1	4	3	1	3	3867.2873	0.0040
4	0	4	3	0	3	3932.3399	0.0104
3	1	2	2	0	2	4265.9482	0.0165
4	2	3	3	2	2	4282.5243	0.0077
4	3	2	3	3	1	4414.7782	-0.0152
4	1	3	3	1	2	4549.9694	0.0030
3	2	1	2	1	1	4561.3469	-0.0018
5	1	5	4	1	4	4781.6424	0.0073
5	0	5	4	0	4	4810.7006	-0.0038
3	2	2	2	1	2	4865.3736	-0.0028
5	2	4	4	2	3	5284.6017	-0.0166
3	3	0	2	2	0	5464.8201	0.0002
3	3	1	2	2	1	5510.1069	0.0006
5	3	3	4	3	2	5515.6536	-0.0026
5	1	4	4	1	3	5523.8147	0.0054
6	1	6	5	1	5	5685.2294	-0.0023
6	0	6	5	0	5	5696.4427	0.0043
4	2	2	3	1	2	5754.4262	-0.0012
4	1	3	3	0	3	5769.1964	-0.0016
5	2	3	4	2	2	5869.0471	0.0053
4	2	3	3	1	3	6213.3795	0.0038
6	2	5	5	2	4	6251.3523	-0.0038
6	1	5	5	1	4	6415.0664	-0.0010
4	3	1	3	2	1	6498.4613	-0.0001
7	1	7	6	1	6	6583.6962	-0.0006
7	0	7	6	0	6	6587.6612	-0.0021
4	3	2	3	2	2	6679.1979	-0.0017
6	4	2	5	4	1	6725.7164	-0.0159
6	2	4	5	2	3	6986.9207	0.0070
5	2	3	4	1	3	7073.5034	0.0007
7	2	6	6	2	5	7188.2262	-0.0022
7	1	6	6	1	5	7275.9865	0.0119
5	1	4	4	0	4	7360.6878	0.0100
4	4	0	3	3	0	7458.1250	-0.0034
4	4	1	3	3	1	7467.6121	-0.0053
8	1	8	7	1	7	7480.0657	-0.0017
5	3	2	4	2	2	7528.5444	0.0050
5	3	3	4	2	3	7912.3359	-0.0032

Table S19. Measured frequencies and residuals (in MHz) for the rotational transitions of conformer **VI**.

J’	K'_{-1}	K'_{+1}	J”	K''_{-1}	K''_{+1}	ν_{obs}	ν_{obs}-ν_{calc}
2	1	1	1	0	1	2948.8703	-0.0065
2	2	1	1	1	1	3423.5417	-0.0056
3	1	2	2	0	2	4436.8711	-0.0018
3	2	1	2	1	1	4519.2404	0.0035
3	2	2	2	1	2	4779.3145	-0.0035
3	3	0	2	2	0	5121.7544	-0.0088
3	3	1	2	2	1	5194.1657	-0.0092
4	2	2	3	1	2	5873.2444	0.0026
4	1	3	3	0	3	6038.0664	0.0072
4	2	3	3	1	3	6220.8158	-0.0004
4	3	1	3	2	1	6232.1137	-0.0046
4	3	2	3	2	2	6463.7185	-0.0075
4	4	0	3	3	0	6973.0222	-0.0246
4	4	1	3	3	1	6998.6729	-0.0295
5	2	3	4	1	3	7380.7334	-0.0019
5	3	2	4	2	2	7431.1968	-0.0042
5	1	4	4	0	4	7651.9888	-0.0024
5	2	4	4	1	4	7723.3725	0.0063
5	3	3	4	2	3	7818.6259	-0.0073

Table S20. Measured frequencies and residuals (in MHz) for the rotational transitions of conformer **VII**.

J'	K'_{-1}	K'_{+1}	J"	$K"_{-1}$	$K"_{+1}$	ν_{obs}	ν_{obs}-ν_{calc}
2	1	1	1	0	1	2996.6621	-0.0045
2	2	0	1	1	0	3382.2550	-0.0010
2	2	1	1	1	1	3512.7079	0.0074
3	1	2	2	0	2	4497.5074	-0.0034
3	2	1	2	1	1	4607.7336	0.0050
3	2	2	2	1	2	4882.0376	0.0123
3	3	0	2	2	0	5266.5454	0.0056
3	3	1	2	2	1	5336.2165	0.0042
4	2	2	3	1	2	5964.0293	-0.0003
4	1	3	3	0	3	6119.9328	0.0039
4	2	3	3	1	3	6338.4500	0.0011
4	3	1	3	2	1	6382.7769	0.0056
4	3	2	3	2	2	6614.8154	0.0000
4	4	0	3	3	0	7173.5053	-0.0071
4	4	1	3	3	1	7196.3145	-0.0046
5	2	3	4	1	3	7474.1683	0.0025
5	3	2	4	2	2	7574.8089	-0.0043
5	1	4	4	0	4	7767.3184	-0.0024
5	3	3	4	2	3	7977.7700	-0.0099

Table S21. Benchmarking of the theoretical rotational constants against the experimental (A_{calc} − A_{exp})/A_{exp} × 100%.

		**Exp.**	**B3LYP**	**% error**	**MP2**	**% error**
**I**	**A**	865.6533	872.3	0.8%	888.5	2.6%
	**B**	700.4774	691.1	-1.3%	693.2	-1.0%
	**C**	449.1701	447.0	-0.5%	453.0	0.9%
**II**	**A**	894.3034	896.1	0.2%	918.6	2.7%
	**B**	677.7762	693.2	2.3%	697.0	2.8%
	**C**	451.3106	450.7	-0.1%	459.1	1.7%
**III**	**A**	966.1056	967.3	0.1%	979.6	1.4%
	**B**	638.5465	632.2	-1.0%	641.2	0.4%
	**C**	453.9216	450.4	-0.8%	459.4	1.2%
**IV**	**A**	868.6112	858.0	-1.2%	880.8	1.4%
	**B**	739.8331	740.9	0.1%	741.3	0.2%
	**C**	463.7072	461.2	-0.5%	468.1	0.9%
**V**	**A**	987.5630	988.5	0.1%	1002.0	1.5%
	**B**	634.2307	630.1	-0.7%	635.2	0.2%
	**C**	447.6706	445.6	-0.5%	451.7	0.9%
**VI**	**A**	915.2201	914.3	-0.1%	925.0	1.1%
	**B**	677.8858	673.7	-0.6%	680.3	0.4%
	**C**	444.2806	442.3	-0.4%	447.9	0.8%
**VII**	**A**	942.6801	943.7	0.1%	951.8	1.0%
	**B**	684.6639	682.2	-0.4%	687.6	0.4%
	**C**	454.1837	453.3	-0.2%	458.3	0.9%

Table S22. Experimental spectroscopic parameters for all singly substituted ^{13}C isotopic species of conformer **I** of cyclododecanone.

**Parameter**	**^{13}C1**	**^{13}C2**	**^{13}C3**	**^{13}C4**	**^{13}C5**	**^{13}C6**
A^a (MHz)	864.87370(50)[d]	861.13503(64)	859.18593(64)	858.93471(64)	859.15910(84)	863.74937(65)
B (MHz)	694.12935(43)	695.25709(57)	699.31745(54)	700.26558(56)	697.84486(66)	695.85541(54)
C (MHz)	446.6440(38)	445.8214(48)	447.2179(50)	447.4311(58)	446.3518(71)	447.2838(47)
σ^b (kHz)	4.6	5.8	5.3	5.8	6.2	5.8
N[c]	12	13	14	13	11	14

**Parameter**	**^{13}C7**	**^{13}C8**	**^{13}C9**	**^{13}C10**	**^{13}C11**	**^{13}C12**
A (MHz)	865.45973(57)	864.63984(57)	859.48402(70)	859.11877(80)	858.85381(60)	860.93795(55)
B (MHz)	696.17817(47)	694.31823(47)	696.61313(72)	700.06292(55)	699.71525(54)	696.70868(47)
C (MHz)	447.3455(44)	446.4771(41)	445.9561(64)	447.4101(55)	447.5108(52)	446.5277(42)
σ (kHz)	4.5	5.1	6.1	4.5	5.4	5.1
N	11	12	13	10	13	15

[a] A, B and C are the rotational constants. Δ_J, Δ_{JK}, Δ_K, δ_J and δ_K centrifugal distortion constants were fixed to parent species values. [b] σ is the rms deviation of the fit. [c] N is the number of the fitted transitions. [d] Standard error in parentheses in units of the last digit.

Table S23. Substitution coordinates of the heavy atoms of conformer **I** of cyclododecanone in Å.

	a	***b***	***c***
C_1	2.4947(14)[a]	-0.3931(91)	0.6193(57)
C_2	2.3021(17)	-1.7832(23)	-0.100(40)
C_3	0.9169(37)	-2.0230(17)	-0.5807(60)
C_4	-0.144(24)	-2.0919(17)	0.4476(81)
C_5	-1.6219(29)	-2.1208(23)	-0.105(46)
C_6	-2.0287(16)	-0.8018(41)	-0.8186(40)
C_7	-2.1138(15)	0.3760(87)	0.000(40)[b]
C_8	2.4986(13)	0.7512(44)	-0.3839(84)
C_9	1.9629(22)	2.0689(22)	0.198(23)
C_{10}	0.4537(93)	2.0585(21)	0.4678(94)
C_{11}	-0.4142(85)	2.0047(18)	-0.7860(47)
C_{12}	-1.9010(18)	1.7509(20)	-0.4690(75)

[a] The errors include Costain's error. [b] Imaginary coordinate set to zero.

Table S24. The Cartesian coordinates of the conformer **I** of cyclododecanone from B3LYP-D3BJ/3-611++G(d,p).

Centre number	Atomic Number	Coordinates (Å)		
		X	Y	Z
1	6	-2.477102	0.473776	0.595390
2	6	-2.219709	1.839251	-0.060884
3	6	-0.794690	2.011193	-0.611634
4	6	0.287131	2.077147	0.472622
5	6	1.726226	2.073248	-0.062762
6	6	2.128470	0.764810	-0.752025
7	6	2.168120	-0.443034	0.177685
8	8	2.402531	-0.323466	1.362264
9	6	-2.497041	-0.703266	-0.398968
10	6	-1.985902	-2.028011	0.188259
11	6	-0.479885	-2.039958	0.493215
12	6	0.401886	-1.997401	-0.759588
13	6	1.901081	-1.799833	-0.449957
14	1	-1.718883	0.301438	1.364077
15	1	-3.431469	0.504772	1.130925
16	1	-2.939769	1.985736	-0.874717
17	1	-2.416071	2.633876	0.668397
18	1	-3.518420	-0.842823	-0.767687
19	1	-1.903505	-0.452524	-1.282980
20	1	-0.578278	1.192805	-1.305443
21	1	-0.749862	2.926956	-1.212547
22	1	-2.537131	-2.246231	1.110186
23	1	-2.216452	-2.845170	-0.505781
24	1	0.134209	2.985729	1.065742
25	1	0.178419	1.244597	1.172236
26	1	-0.228136	-1.200972	1.147737
27	1	-0.232244	-2.940115	1.066100
28	1	2.413604	2.248969	0.767966
29	1	1.860343	2.898833	-0.770594
30	1	0.281433	-2.929636	-1.321099
31	1	0.068881	-1.198949	-1.428610
32	1	1.491265	0.546967	-1.612707
33	1	3.145478	0.854421	-1.157825
34	1	2.251953	-2.568962	0.241650
35	1	2.472888	-1.879525	-1.381256

Table S25. The Cartesian coordinates of the conformer **II** of cyclododecanone from B3LYP-D3BJ/3-611++G(d,p).

Centre number	Atomic Number	Coordinates (Å)		
		X	Y	Z
1	6	-2.587084	-1.240685	0.554532
2	6	-1.445320	-2.079573	-0.054339
3	6	-0.059429	-1.528918	0.287638
4	6	1.129844	-2.259188	-0.331021
5	6	2.473237	-1.587799	-0.005946
6	6	2.622214	-0.169940	-0.589578
7	6	1.826595	0.904277	0.151584
8	8	1.877792	0.998261	1.359779
9	6	-3.052415	-0.026834	-0.274499
10	6	-1.983775	0.994084	-0.695713
11	6	-1.273719	1.708702	0.461549
12	6	-0.108106	2.613253	0.027456
13	6	1.030333	1.886901	-0.699222
14	1	-3.462845	-1.881357	0.700734
15	1	-2.288818	-0.916341	1.557966
16	1	-1.567241	-2.132732	-1.144209
17	1	-1.519491	-3.110710	0.309372
18	1	-3.836039	0.491446	0.291597
19	1	-3.536110	-0.401592	-1.184621
20	1	0.061240	-1.504413	1.376782
21	1	-0.025564	-0.490523	-0.028803
22	1	-2.460368	1.747784	-1.334390
23	1	-1.256132	0.489087	-1.338277
24	1	1.004399	-2.310153	-1.420786
25	1	1.154707	-3.296076	0.023448
26	1	-2.006748	2.322409	0.997326
27	1	-0.903981	0.984796	1.190335
28	1	3.287562	-2.205938	-0.395427
29	1	2.609053	-1.529832	1.078315
30	1	-0.486668	3.400207	-0.634543
31	1	0.301836	3.109624	0.910426
32	1	2.365513	-0.167212	-1.653149
33	1	3.672307	0.136463	-0.514464
34	1	1.764546	2.618933	-1.064751
35	1	0.672247	1.374363	-1.595769

Table S26. The Cartesian coordinates of the conformer **III** of cyclododecanone from B3LYP-D3BJ/3-611++G(d,p).

Centre number	Atomic Number	Coordinates (Å)		
		X	Y	Z
1	6	-2.958544	0.942665	-0.085280
2	6	-2.731305	-0.476782	-0.639921
3	6	-2.243016	-1.537281	0.367658
4	6	-0.729601	-1.601147	0.641178
5	6	0.110509	-2.003086	-0.575370
6	6	1.627361	-2.021345	-0.292711
7	6	2.171303	-0.667481	0.135117
8	8	2.560090	-0.470258	1.267663
9	6	-1.749175	1.625785	0.569088
10	6	-0.520726	1.739300	-0.340137
11	6	0.765608	2.070325	0.423767
12	6	2.060548	1.853230	-0.373506
13	6	2.229760	0.428271	-0.922901
14	1	-3.324636	1.571478	-0.906119
15	1	-3.771601	0.901201	0.649314
16	1	-2.053138	-0.436599	-1.497783
17	1	-3.688627	-0.815743	-1.049530
18	1	-2.046855	2.622928	0.912553
19	1	-1.472469	1.074836	1.474055
20	1	-2.557731	-2.524304	0.008225
21	1	-2.766143	-1.386179	1.319297
22	1	-0.389737	0.791890	-0.864377
23	1	-0.701275	2.486539	-1.122760
24	1	-0.551445	-2.330321	1.439645
25	1	-0.371807	-0.648637	1.034440
26	1	0.732183	3.108599	0.771417
27	1	0.812300	1.453409	1.325929
28	1	-0.092146	-1.332983	-1.415553
29	1	-0.188666	-3.001901	-0.910930
30	1	2.910383	2.083443	0.273533
31	1	2.104630	2.550873	-1.217310
32	1	1.857146	-2.733904	0.502530
33	1	2.154701	-2.336103	-1.201094
34	1	1.509741	0.216392	-1.716480
35	1	3.218146	0.332425	-1.392201

Table S27. The Cartesian coordinates of the conformer **IV** of cyclododecanone from B3LYP-D3BJ/3-611++G(d,p).

Centre number	Atomic Number	Coordinates (Å)		
		X	Y	Z
1	6	-2.544407	-0.838240	-0.250545
2	6	-2.578043	0.535522	0.458518
3	6	-2.203144	1.713154	-0.454332
4	6	-0.712145	1.737881	-0.823428
5	6	0.186338	2.247629	0.311070
6	6	1.697394	2.022561	0.077596
7	6	2.077791	0.569532	0.322062
8	8	2.280773	0.168712	1.449422
9	6	-1.861340	-1.943429	0.570185
10	6	-0.338237	-1.770855	0.674305
11	6	0.408152	-2.171904	-0.605275
12	6	1.907252	-1.830608	-0.626768
13	6	2.210219	-0.349014	-0.883988
14	1	-3.566512	-1.144057	-0.493802
15	1	-2.032242	-0.744444	-1.214032
16	1	-1.901387	0.522675	1.318855
17	1	-3.575621	0.704137	0.874978
18	1	-2.297168	-1.955855	1.575757
19	1	-2.084626	-2.922069	0.128859
20	1	-2.804475	1.658491	-1.368902
21	1	-2.469246	2.659722	0.030657
22	1	0.049648	-2.371912	1.502976
23	1	-0.112277	-0.737262	0.942148
24	1	-0.555790	2.371159	-1.703830
25	1	-0.408824	0.731296	-1.122279
26	1	-0.068083	-1.712698	-1.480253
27	1	0.292939	-3.253468	-0.740168
28	1	0.009717	3.319699	0.444194
29	1	-0.082283	1.774371	1.260001
30	1	2.385576	-2.415340	-1.418278
31	1	2.375319	-2.129438	0.315524
32	1	1.969439	2.331489	-0.937017
33	1	2.264530	2.626144	0.789566
34	1	1.610287	0.036853	-1.713245
35	1	3.255915	-0.232564	-1.203481

Table S28. The Cartesian coordinates of the conformer **V** of cyclododecanone from B3LYP-D3BJ/3-611++G(d,p).

Centre number	Atomic Number	Coordinates (Å)		
		X	Y	Z
1	6	-0.971000	2.187479	0.408262
2	6	0.251783	1.686067	-0.365795
3	6	1.553405	1.778349	0.436002
4	6	2.772847	1.124924	-0.238937
5	6	2.947648	-0.389223	-0.024063
6	6	1.900767	-1.297501	-0.681711
7	6	0.645749	-1.561346	0.145220
8	8	0.631639	-1.415423	1.348978
9	6	-2.323648	1.864777	-0.252270
10	6	-2.509212	0.384954	-0.632310
11	6	-2.308794	-0.593500	0.536725
12	6	-1.898244	-2.016516	0.128382
13	6	-0.560842	-2.094360	-0.618307
14	1	-0.945272	1.755440	1.413658
15	1	-0.893687	3.271289	0.551133
16	1	0.355714	2.244547	-1.304982
17	1	0.085227	0.650089	-0.659744
18	1	-3.125623	2.171434	0.429267
19	1	-2.442104	2.473257	-1.156551
20	1	1.772170	2.835822	0.623122
21	1	1.401584	1.317185	1.417947
22	1	-1.812707	0.148912	-1.441584
23	1	-3.508006	0.247237	-1.060773
24	1	3.679150	1.605301	0.144421
25	1	2.753396	1.340334	-1.315634
26	1	-3.231889	-0.646857	1.123753
27	1	-1.545691	-0.217088	1.217536
28	1	2.965129	-0.604469	1.048320
29	1	3.930692	-0.668080	-0.415910
30	1	-2.670111	-2.465202	-0.507293
31	1	-1.833648	-2.631815	1.029651
32	1	2.333478	-2.292402	-0.857839
33	1	1.616773	-0.926598	-1.671593
34	1	-0.611986	-1.588667	-1.586931
35	1	-0.328456	-3.140899	-0.858976

Table S29. The Cartesian coordinates of the conformer **VI** of cyclododecanone from B3LYP-D3BJ/3-611++G(d,p).

Centre number	Atomic Number	Coordinates (Å)		
		X	Y	Z
1	6	0.466745	2.228247	0.457782
2	6	-0.515762	1.888649	-0.668861
3	6	-1.985482	1.822942	-0.220327
4	6	-2.355594	0.646619	0.704381
5	6	-2.741442	-0.670594	0.008935
6	6	-1.643661	-1.346379	-0.819363
7	6	-0.425190	-1.777865	-0.014091
8	8	-0.491588	-1.964882	1.182774
9	6	1.947664	2.134183	0.052498
10	6	2.390976	0.744975	-0.435447
11	6	2.250314	-0.360801	0.622575
12	6	2.138667	-1.783421	0.054929
13	6	0.871428	-2.011926	-0.781281
14	1	0.261007	3.245138	0.811452
15	1	0.283265	1.575481	1.315713
16	1	-0.231337	0.941028	-1.128568
17	1	-0.416803	2.641904	-1.459845
18	1	2.149513	2.866759	-0.738067
19	1	2.565712	2.429589	0.908547
20	1	-2.632146	1.796870	-1.106109
21	1	-2.223206	2.761779	0.293250
22	1	3.433076	0.797385	-0.769086
23	1	1.813791	0.486825	-1.327823
24	1	-3.215006	0.944966	1.313383
25	1	-1.547817	0.451515	1.413680
26	1	3.106363	-0.314659	1.304003
27	1	1.370579	-0.179910	1.242393
28	1	-3.600603	-0.490022	-0.646994
29	1	-3.073555	-1.374930	0.776147
30	1	2.142213	-2.494901	0.884041
31	1	3.011135	-2.011920	-0.567341
32	1	-1.326002	-0.721433	-1.656831
33	1	-2.040686	-2.262989	-1.278456
34	1	0.838266	-3.053868	-1.126985
35	1	0.872976	-1.403320	-1.689080

Table S30. The Cartesian coordinates of the conformer **VII** of cyclododecanone from B3LYP-D3BJ/3-611++G(d,p).

Centre number	Atomic Number	Coordinates (Å)		
		X	Y	Z
1	6	2.544409	-0.838239	-0.250543
2	6	2.578039	0.535524	0.458520
3	6	2.203144	1.713154	-0.454333
4	6	0.712144	1.737883	-0.823430
5	6	-0.186338	2.247627	0.311070
6	6	-1.697394	2.022559	0.077599
7	6	-2.077792	0.569531	0.322062
8	8	-2.280777	0.168710	1.449421
9	6	1.861341	-1.943429	0.570185
10	6	0.338238	-1.770855	0.674304
11	6	-0.408150	-2.171903	-0.605276
12	6	-1.907250	-1.830608	-0.626771
13	6	-2.210218	-0.349014	-0.883989
14	1	2.032249	-0.744445	-1.214033
15	1	3.566517	-1.144054	-0.493795
16	1	3.575615	0.704140	0.874984
17	1	1.901379	0.522677	1.318853
18	1	2.297168	-1.955855	1.575757
19	1	2.084627	-2.922069	0.128859
20	1	2.804476	1.658490	-1.368902
21	1	2.469245	2.659722	0.030656
22	1	-0.049647	-2.371913	1.502975
23	1	0.112279	-0.737262	0.942149
24	1	0.555790	2.371164	-1.703831
25	1	0.408823	0.731299	-1.122284
26	1	-0.292937	-3.253467	-0.740170
27	1	0.068084	-1.712696	-1.480254
28	1	0.082285	1.774366	1.260000
29	1	-0.009718	3.319697	0.444197
30	1	-2.375319	-2.129441	0.315521
31	1	-2.385573	-2.415338	-1.418282
32	1	-2.264528	2.626142	0.789570
33	1	-1.969440	2.331489	-0.937013
34	1	-1.610285	0.036855	-1.713244
35	1	-3.255914	-0.232564	-1.203483
